# Association Between COVID-19 Vaccination and Long COVID Symptoms in Hospitalised Survivors: Distinguishing Prevention from Reverse Causality

**DOI:** 10.3390/biomedicines14020350

**Published:** 2026-02-02

**Authors:** Lanre Peter Daodu, Yogini Raste, Judith E. Allgrove, Francesca I. F. Arrigoni, Reem Kayyali

**Affiliations:** 1 Faculty of Health, Science, Social Care and Education, Kingston University, London KT1 2EE, UK; f.arrigoni@kingston.ac.uk (F.I.F.A.); r.kayyali@kingston.ac.uk (R.K.); 2 Chest/Respiratory Department, Croydon University Hospital, Croydon Health Services NHS Trust, London CR7 7YE, UK; yoginiraste@nhs.net; 3 School of Allied Health and Exercise Sciences, Faculty of Health, Environment and Medical Sciences, Bournemouth University, Bournemouth BH8 8GP, UK; jallgrove@bournemouth.ac.uk

**Keywords:** long COVID, COVID-19 vaccination, reverse causality, post-acute sequelae of SARS-CoV-2 (PASC), Bayesian analysis, heterologous vaccination

## Abstract

**Background**: While COVID-19 vaccination significantly reduces acute disease severity, its impact on the incidence of long COVID remains debated, with some observational studies paradoxically suggesting higher symptom rates among vaccinated individuals. This study aimed to resolve this controversy by distinguishing between the protective effects of prior immunity and the confounding influence of reverse causality. **Methods**: We conducted a retrospective cohort study of 627 adults hospitalised for COVID-19 in London. Participants were stratified into two analytical cohorts based on vaccination timing: a “prevention cohort” (vaccinated ≥14 days pre-infection) and a “post-acute cohort” (vaccinated post-infection). Multivariable Bayesian logistic regression was employed to estimate Adjusted Odds Ratios (aOR) for long COVID, controlling for age, gender, BMI, comorbidities, and acute length of hospital stay (LoS). **Results**: In the prevention cohort, prior vaccination demonstrated a non-significant protective trend against long COVID (aOR 0.81; 95% CI 0.45–1.42; *p* = 0.45), with no significant difference observed between homologous and heterologous regimens. The post-acute cohort exhibited a strong, significant positive association (aOR 3.41; 95% CI 2.23–5.52; *p* < 0.001), indicating substantial indication bias, with symptomatic individuals more likely to seek vaccination. The strongest independent predictors of long COVID were comorbidities (aOR 2.78) and prolonged acute hospitalisation (≥4 days; aOR 1.82). **Conclusions**: Vaccination administered prior to infection demonstrates a protective trend against long COVID, whereas the strong association observed with post-infection vaccination reflects indication bias, with symptomatic survivors being more likely to seek immunisation. Clinical strategies to mitigate post-acute sequelae should prioritise reducing acute disease severity and managing comorbidities, which were identified as the dominant independent predictors of risk in hospitalised patients.

## 1. Background

Since its emergence, the COVID-19 pandemic has transitioned from an acute global emergency to a persistent public health challenge, defined mainly by the enduring burden of Post-Acute Sequelae of SARS-CoV-2 (PASC), commonly known as long COVID. Characterised by a constellation of debilitating symptoms, including fatigue, dyspnoea, and cognitive dysfunction, that persist for months or years after the initial infection, long COVID affects an estimated 10–20% of survivors [1,2]. As the virus becomes endemic, understanding modifiable risk factors for these sequelae is essential for reducing the long-term burden on healthcare systems.

The rapid development and deployment of COVID-19 vaccines, utilising novel mRNA and adenoviral vector technologies, proved highly effective in preventing hospitalisation and mortality during the acute phase of the pandemic [3]. However, the extent to which vaccination protects against the downstream development of long COVID remains a subject of debate. Large-scale registry studies suggest that vaccination prior to infection offers a partial protective effect, reducing the risk of long COVID by approximately 15–50% [4,5,6]. Biological plausibility for this protection exists; pre-existing immunity may accelerate viral clearance and reduce the systemic inflammation associated with PASC [1]. Conversely, other observational studies have reported negligible benefits or, paradoxically, higher rates of long COVID symptoms among vaccinated cohorts [5].

These conflicting findings are likely driven by methodological limitations inherent to observational research, specifically temporal ambiguity and indication bias. In real-world datasets, “vaccinated” cohorts often include individuals immunised before infection, as well as those who sought vaccination after recovering from the acute phase. This conflation introduces reverse causality, where individuals with lingering symptoms are more likely to seek vaccination as a therapeutic measure, thereby artificially inflating the apparent risk in the vaccinated group [3,5]. Furthermore, while immunological data suggest that heterologous vaccine regimens may induce broader neutralising antibody responses than homologous regimens [7,8], it remains unclear whether this translates into superior clinical protection against long COVID.

This study aims to resolve these inconsistencies by analysing electronic health records from a cohort of adults hospitalised with COVID-19 in London. Unlike previous studies that pooled vaccination status, we employ a rigorous temporal stratification to distinguish between the preventative effects of pre-infection immunity and the health-seeking behaviours associated with post-infection vaccination. Utilising Bayesian logistic regression to handle small subgroups and potential data separation, we sought to (1) determine whether vaccination prior to infection reduces the odds of long COVID in a high-severity cohort; (2) quantify the extent of reverse causality by analysing post-infection vaccination patterns; and (3) investigate whether heterologous vaccine regimens offer superior protection compared to homologous regimens.

## 2. Materials and Methods

### 2.1. Study Design and Data Source

This retrospective observational cohort study was conducted at a single university hospital in London, United Kingdom. We analysed anonymised Electronic Patient Records (EPR) of adults diagnosed with COVID-19 between April 2020 and December 2022. Data entry and management were facilitated using Castor Electronic Data Capture (EDC) software v2021.4, AMS, NLand the study was reported according to the Strengthening the Reporting of Observational Studies in Epidemiology (STROBE) guidelines. 

Ethical approval for the research was obtained from the Health Research Authority (HRA) England and Health and Care Research Wales (HCRW), under REC reference 23/HRA/1637.

### 2.2. Study Population and Eligibility

The study included adult patients (≥18 years) with a confirmed SARS-CoV-2 infection by PCR or rapid antigen testing who required hospital admission during the acute phase of illness. Participants were excluded from the study if they lacked valid dates for either infection, hospital admission, or vaccination. Additionally, individuals who received their first vaccine dose within 0 to 13 days before infection were excluded. This criterion was implemented to eliminate cases with ambiguous immune status, specifically those in which the vaccine had been administered but had not yet provided biological protection, or in which the participant might have been incubating the virus at the time of vaccination.

### 2.3. Variable Definitions and Data Engineering

#### 2.3.1. Outcome Variable

The primary outcome was long COVID, defined as a binary variable (Yes/No). This was determined based on clinician-documented diagnosis or patient-reported persistence of symptoms such as fatigue, dyspnoea, and cognitive dysfunction (brain fog) beyond 12 weeks post-infection, consistent with the World Health Organisation (WHO) clinical case definition. Where available, specific ICD-10 codes associated with post-COVID conditions, such as U09.9 “Post COVID-19 condition, unspecified,” were used to support classification. The combination of symptom duration, clinical descriptors, and ICD coding ensured consistent and reproducible identification of long COVID cases for analysis and comparison with other studies.

#### 2.3.2. Exposure Variables and Cohort Stratification

To account for reverse causality, where symptomatic individuals may be more likely to seek vaccination, and to minimise immortal time bias, the participants were not analysed as a single pooled group. Instead, we divided the study population into two analytical cohorts based on the timing of vaccination relative to infection. The first cohort was the prevention analysis (Analysis A), which compared individuals who had been vaccinated at least 14 days before their initial confirmed COVID-19 infection with those who remained unvaccinated, thereby assessing the biological protective effect of prior immunity. The second cohort was the post-acute analysis (Analysis B), which compared individuals who received vaccination after their acute infection with unvaccinated individuals to examine health-seeking behaviours and the relationship between persistent symptoms and subsequent vaccine uptake. In the prevention cohort, vaccinated participants were further classified by vaccine regimen (homologous or heterologous) to evaluate potential differences in protective effect. The heterologous regimens were defined as those in which participants received COVID-19 vaccines from two or more different platforms, including mRNA-based vaccines (such as Pfizer–BioNTech BNT162b2 or Moderna mRNA-1273), adenoviral vector vaccines (such as Oxford-AstraZeneca ChAdOx1 nCoV-19), or protein subunit vaccines. In contrast, homologous regimens comprised participants who received all vaccine doses from the same platform, regardless of manufacturer. Assignment to the heterologous or homologous groups was based on documented vaccine brand information in the EPR.

#### 2.3.3. Covariates and Justification for Inclusion/Exclusion

To estimate the independent effect of vaccination while controlling for confounding factors, several covariates were engineered and included in the final models. Acute severity was assessed using the length of hospital stay (LoS), calculated as the number of days between admission and discharge, since all participants were hospitalised and a binary hospitalisation status variable would not provide statistical variance. To address convergence issues caused by extreme outliers, such as stays longer than 100 days, the length of hospital stay was dichotomised into short (<4 days) and long (≥4 days) based on the cohort median. This dichotomised measure served as the primary proxy for acute disease severity. The initial comorbidity count, ranging from 0 to 12, yielded sparse data; for example, no unvaccinated patients had 11 comorbidities, rendering the model unstable. As a result, comorbidities were transformed into a binary variable, distinguishing between none (zero comorbidity) and any (one or more comorbidities).

Standard demographic confounders were also included in the analysis: age and body mass index (BMI), both as continuous numeric variables, and gender as a categorical factor. Smoking status and ethnicity were initially included in exploratory models; however, these variables resulted in “perfect separation,” with zero event counts in specific subgroups. This issue arose due to the relatively modest sample size. To maintain model stability and ensure sufficient statistical power to assess the primary exposure of interest, which was vaccination, these covariates were excluded from the final adjusted model.

### 2.4. Statistical Analysis

Data analysis was conducted using R Statistical Software (version 4.5.2). Continuous variables were summarised using means and standard deviations (SD), while categorical variables were presented as frequencies and percentages. Differences between groups were assessed using the Wilcoxon rank-sum test for continuous variables and Chi-square or Fisher’s exact tests for categorical variables, as appropriate.

#### 2.4.1. Outcome Modelling Strategy

To evaluate the association between vaccination and long COVID, we employed multivariable Bayesian logistic regression using the bayesglm function from the arm package. This method was selected over standard maximum likelihood estimation (GLM) and Propensity Score Weighting to address issues of perfect separation (zero cell counts) and small sample sizes within specific subgroups, such as particular comorbidity profiles or extreme LoS. The Bayesian approach uses weakly informative priors to stabilise coefficient estimates, yielding robust Adjusted Odds Ratios (aORs) and 95% Confidence Intervals (CIs).

#### 2.4.2. Primary Cohort Analysis

Two distinct multivariable models were developed to separate the preventative effect of vaccination from the possibility of reverse causality. The first, the prevention model, assessed the odds of developing long COVID among individuals who had received a vaccine at least 14 days before their initial infection, compared with those who remained unvaccinated. The second was the post-acute model, which examined the odds of long COVID in participants who were vaccinated only after their infection had begun, again using the unvaccinated group as the baseline for comparison.

#### 2.4.3. Subgroup Analysis: Vaccine Regimen

To investigate whether heterologous vaccination provided superior protection compared with homologous regimens, a subgroup analysis was performed within the prevention cohort. The exposure variable was categorised into three levels: unvaccinated (reference group), homologous (received the same vaccine type/brand), and heterologous (received different vaccine types/brands). This model assessed whether the heterologous and homologous groups differed significantly from the unvaccinated baseline or from each other, while adjusting for the same set of covariates.

#### 2.4.4. Model Adjustments

All regression models were adjusted to account for several potential confounding variables. Specifically, demographic factors included age (continuous), gender (male or female), and body mass index (BMI; continuous). Clinical status was represented by the presence of comorbidities, coded as a binary variable distinguishing between individuals with no comorbidities and those with at least one comorbidity. The severity of the acute phase was controlled for by including the LoS, classified as short (fewer than 4 days) or long (4 days or more). Statistical significance was determined by a 95% confidence interval that did not include 1.0, and all *p*-values were two-tailed.

## 3. Results

### 3.1. Characteristics of the Study Participants

The study cohort consisted of 627 adult participants hospitalised with acute COVID-19; the demographic and clinical characteristics are presented in Table 1. The mean age of participants was 59 years (SD ± 20), with a slightly higher prevalence of women (55%, *n* = 344) compared to men (45%, *n* = 283). The cohort had a mean Body Mass Index (BMI) of 29 kg/m^2^ (SD ± 7), within the overweight-to-obese range. The study population was ethnically diverse: 41% identified as White, followed by Black, African, Caribbean, or British (23%), and Asian or Asian British (19%). In relation to smoking history, most participants (62%) were non-smokers, while 13% were former smokers and 8.8% were current smokers. 63% (*n* = 393) had received at least one dose of a COVID-19 vaccine, while 37% (*n* = 234) remained unvaccinated. Among those who were vaccinated, the timing of the first dose varied considerably: 42% (167 individuals) received their first dose before contracting COVID-19 (the prevention cohort), while 58% (226 individuals) received it after the onset of acute infection (the post-acute cohort). The most common number of doses was three (29%), followed by four (18%) and two (13%). Among vaccinated individuals, the majority (63%) followed a homologous regimen, while 37% received a heterologous regimen. Long COVID symptoms lasting beyond 12 weeks were reported by 40% (252 individuals) of the total study population.

### 3.2. Primary Analysis: Association Between Prior Vaccination and Long COVID

In the prevention cohort, after adjusting for age, gender, BMI, comorbidities, and LoS, vaccination prior to infection showed a protective, but non-statistically significant trend against developing long COVID, [aOR: 0.81 (95% CI: 0.45–1.42, *p* = 0.45)] in Table 2.

### 3.3. Subgroup Analysis: Homologous vs. Heterologous Strategies

Both vaccination strategies, homologous [aOR: 0.76 (95% CI: 0.37–1.47; *p* = 0.41)] and heterologous [aOR: 0.88 (95% CI: 0.41–1.80; *p* = 0.73)] revealed non-statistically significant protective trends compared to the unvaccinated baseline (Table 3).

### 3.4. Secondary Analysis: Reverse Causality (Post-Acute Vaccination)

**Figure 1:**
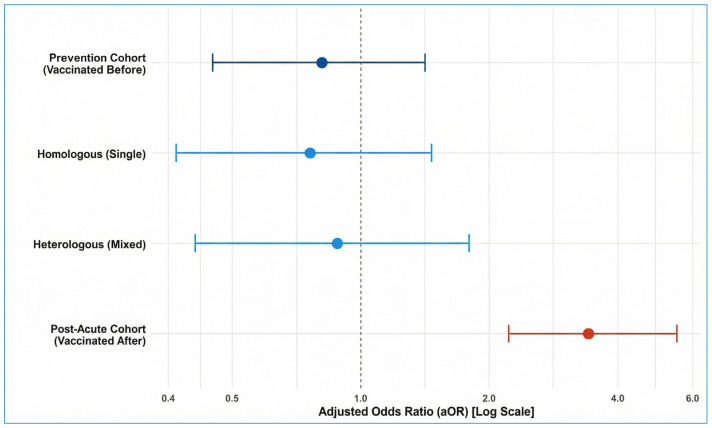
Association Between Vaccination Timing and long COVID. Forest plot displaying Adjusted Odds Ratios (aOR) and 95% Confidence Intervals derived from Bayesian logistic regression models. Dark blue markers (Prevention Cohort): Represent participants vaccinated ≥14 days prior to infection. These estimates show a non-significant trend toward protection (aOR < 1.0). Light Blue markers: Represent exploratory subgroups within the Prevention Cohort, stratified by vaccine regimen (Homologous vs. Heterologous). Both show similar protective trends. Orange marker (Post-Acute Cohort): Represents participants who received their first vaccine dose after the onset of acute infection. This group shows a statistically significant positive association (aOR 3.41), indicative of reverse causality (symptomatic health-seeking behaviour). The vertical dashed line at 1.0 represents no effect. All models were adjusted for age, gender, BMI, comorbidities, and acute length of hospital stay.

**Figure 2:**
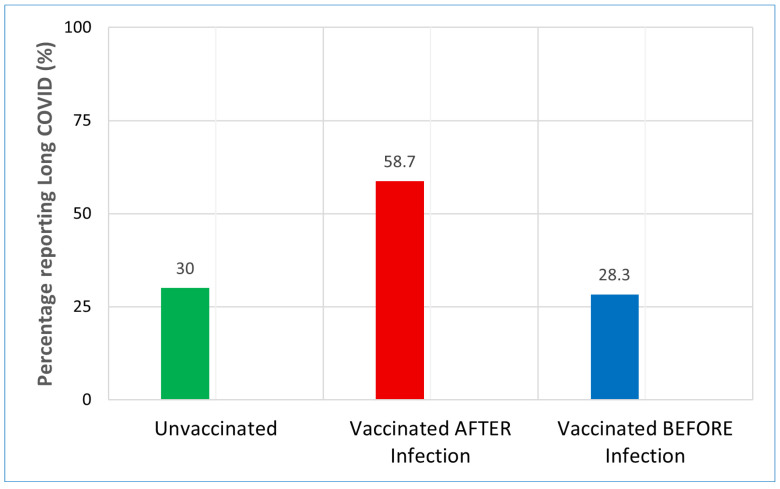
Unadjusted Prevalence of long COVID by Vaccination Timing. Bar chart displaying the raw percentage of participants reporting long COVID symptoms in each cohort, who met the clinical criteria for long COVID (symptoms persisting ≥12 weeks post-infection). Unvaccinated (Green): Baseline symptom prevalence was 30%. Vaccinated BEFORE Infection (Blue): Participants vaccinated before infection showed a slightly lower prevalence (28.3%), consistent with the protective trend observed in the adjusted regression models. Vaccinated AFTER Infection (Red): Participants who received their first dose post-infection had a markedly higher symptom prevalence (58.7%). This disparity supports the hypothesis of reverse causality, in which individuals with persistent symptoms were more likely to seek vaccination than those who had fully recovered.

In post-acute analysis (Figure 1), receiving the first vaccine dose after infection [aOR 3.41; (95% CI: 2.23–5.52; *p* < 0.001)] was strongly and statistically significantly associated with reporting long COVID symptoms. Figure 2 illustrates a marked disparity in the proportion of participants reporting symptoms that persisted for ≥12 weeks. The raw prevalence of long COVID was more than double in the ‘post-acute’ cohort (58.7%) compared to the ‘prevention’ cohort (28.3%).

### 3.5. Independent Predictors of Long COVID

Across all models, the strongest independent predictors of long COVID were clinical indicators of baseline health and acute severity (Table 2). Participants with one or more pre-existing comorbidities had nearly three times the odds of developing long COVID (aOR 2.78). A longer hospital stay (≥4 days), a proxy for acute disease severity, was associated with an 82% increase in the odds of long COVID (aOR 1.82). Older age remained a consistent risk factor, with the odds of long COVID increasing by approximately 5% for every additional year of age. Gender and BMI were not statistically significant predictors in the adjusted models.

## 4. Discussion

This retrospective cohort study of 627 hospitalised adults resolves a critical epidemiological paradox by distinguishing the temporal effects of vaccination on long COVID. By stratifying participants into distinct prevention and post-acute cohorts, the study disentangles the biological protective effects of vaccination from behavioural confounders. The primary finding is that while vaccination administered prior to infection shows a protective trend against long COVID (aOR 0.81), the elevated odds observed with post-infection vaccination (aOR 3.41) are attributable to reverse causality, in which patients with persistent symptoms are significantly more likely to seek vaccination after their acute infection.

Our observation that pre-infection vaccination is associated with a reduction in the odds of long COVID aligns with large-scale epidemiological evidence. Registry studies, such as those involving U.S. Veterans Affairs data [4], UK community cohorts by the Office for National Statistics [5], and other studies [9,10,11], have reported risk reductions ranging from 15% to 50%. Our adjusted odds ratio of 0.81 falls squarely within the 15–41% protective range identified in systematic reviews [6]. However, our study extends this work by resolving the “paradox” of higher vaccination rates among long COVID patients; while unadjusted comparisons in our dataset initially mirrored conflicting reports, our stratified analysis confirms that these associations disappear when proper temporal sequencing is applied, validating the principles of causal inference described by Hernán and Robins [12]. Crucially, our study differs from population-level studies by focusing exclusively on hospitalised survivors. For the vaccinated individuals in our prevention cohort, these represent ‘breakthrough’ severe infections. Observing a protective trend (aOR 0.81) even within this high-severity group suggests that vaccination may offer a ‘protective floor’ against downstream sequelae, even when it fails to prevent the acute hospitalisation itself.

The lack of statistical significance for the protective effect in the prevention cohort (aOR 0.81) may plausibly reflect effect modification by baseline disease severity. In a population already sufficiently ill to require hospital admission, the dominant drivers of long COVID risk—specifically comorbidity burden (aOR 2.78) and prolonged LoS (aOR 1.82)—likely overwhelm the marginal downstream protective effect of vaccination. Consequently, the vaccine’s ability to modulate post-acute sequelae may be harder to detect in this high-severity context compared to community populations where it prevents the initial cascade of severe disease entirely.

A notable and novel finding in our study is the lack of significant difference between homologous and heterologous vaccine regimens in preventing long COVID symptoms. While early immunological studies suggested that heterologous boosting might induce superior broad-spectrum immunity [7,8], our clinical data indicate that for the specific outcome of long COVID prevention, the timing of immunity, established before infection, is more critical than the platform combination used. This suggests that the complexity of the vaccine schedule is less important than the binary state of being immunised prior to viral exposure. However, given the limited sample sizes within these specific regimen subgroups, these findings should be interpreted with caution and viewed as hypothesis-generating rather than definitive evidence of equivalence.

The observed protective trend in the prevention cohort supports the biological hypothesis that pre-existing adaptive immunity facilitates rapid viral clearance, thereby limiting the viral persistence and tissue damage implicated in long COVID pathogenesis [13,14]. Pre-existing immunity likely primes the adaptive immune response to clear viral reservoirs faster, potentially reducing the inflammatory cascade associated with post-acute sequelae. This biological mechanism stands in stark contrast to the findings in our post-acute cohort, which require a behavioural rather than biological explanation.

The strong association observed in the post-acute cohort (aOR 3.41) highlights the phenomenon of indication bias. Patients suffering from debilitating sequelae likely view vaccination as a necessary therapeutic intervention or seek it urgently to prevent reinfection, given their perceived vulnerability [3]. This mirrors patterns observed in other post-viral syndromes, in which symptomatic individuals interact more frequently with healthcare systems. Consequently, the “risk” observed in cross-sectional studies is a marker of health-seeking behaviour, distinguishing it fundamentally from the protective signal observed when vaccination precedes infection.

Beyond vaccination status, the identification of acute disease severity (LoS ≥ 4 days) as a dominant predictor of long COVID (aOR 1.82) has practical diagnostic implications. Clinicians should recognise that hospitalised patients requiring supplemental oxygen or extended admissions are at elevated risk for post-acute sequelae regardless of their vaccination status. Consequently, discharge planning for these high-severity patients should proactively include screening for functional limitations and fatigue at follow-up intervals, rather than waiting for patient self-report [15].

For policymakers, these findings reinforce the imperative to maintain high vaccination coverage, particularly among populations with comorbidities, as preventing severe acute disease is the most effective lever for reducing downstream long COVID prevalence [3]. Operationally, health systems should not prioritise complex heterologous vaccine supply chains solely for long COVID prevention, as standard homologous schedules appear equally effective. Stakeholders can immediately use these data to counter vaccine hesitancy narratives that falsely claim vaccines cause long COVID, focusing instead on the reduction in acute severity as the primary preventative mechanism.

The strength of our interpretation relies on the use of Bayesian logistic regression, which resolved issues of quasi-complete separation arising from zero event counts in specific subgroups, a methodological limitation that caused standard maximum likelihood models to fail. Furthermore, our use of LoS as a proxy for acute severity allowed us to adjust for the most significant confounder in hospitalised populations [3]. This analytic approach increases confidence that the null findings in the prevention cohort are due to statistical power limitations rather than methodological bias. Despite these strengths, we must acknowledge that our single-centre retrospective design limits the sample size, resulting in wide confidence intervals that prevented the protective trend in the prevention cohort from reaching statistical significance. Selection bias is inherent to the study, as the cohort consisted entirely of hospitalised patients, meaning our results cannot be generalised to the majority of long COVID sufferers who had mild acute infections. Consequently, our estimates likely differ from community-based studies where the vaccine’s ability to prevent infection entirely plays a larger role. Our findings represent the impact of vaccination on severe disease phenotypes and may not generalise to mild, non-hospitalised cases of COVID-19, which constitute the majority of long COVID patients globally. Additionally, reliance on electronic health records may introduce measurement bias, as symptoms are recorded only when patients report them to clinicians, potentially underestimating prevalence compared with prospective symptom diaries.

## 5. Conclusions

In this retrospective cohort study, vaccination administered prior to infection was associated with a non-significant protective trend against long COVID, whereas vaccination administered post-infection was strongly associated with increased symptom reporting. This divergence provides critical empirical evidence that previously reported “risks” of vaccination are attributable to reverse causality and indication bias—specifically, symptomatic survivors seeking vaccination—rather than biological harm. Our findings reinforce the established efficacy of COVID-19 vaccination, demonstrating that long-term complications do not offset its acute benefits. While temporal stratification strengthens our inference, residual confounding inherent to observational methodology cannot be fully excluded. Consequently, clinical attention should shift away from unfounded concerns regarding vaccine safety and toward managing the independent predictors of sequelae identified here: comorbidities and acute severity. Specifically, patients with acute hospital stays exceeding four days warrant targeted longitudinal monitoring for functional decline, regardless of their vaccination history. From a policy perspective, maintaining high vaccine coverage in comorbid populations remains the most viable strategy to reduce long COVID incidence by mitigating its primary driver—severe acute disease. Future research should utilise large-scale prospective registries to validate these trends across emerging variants, but current evidence supports the continued prioritisation of vaccination and acute severity reduction as the foundation for minimising the pandemic’s long-term burden.

## Figures and Tables

**Table 1:** Characteristics of the study participants.

Characteristic	*N* = 627
Age, Mean (SD)	59 (20)
Gender, *n* (%)	
Female	344 (55%)
Male	283 (45%)
BMI (kg/m^2^), Mean (SD)	29 (7)
Ethnicity, *n* (%)	
White	256 (41%)
Black, Black British, Caribbean, or African	145 (23%)
Asian or Asian British	118 (19%)
Mixed or multiple ethnic groups	18 (2.9%)
Any other ethnic group	90 (14%)
Smoking Status, *n* (%) ^a^	
Non-smokers	390 (62%)
Former smokers	80 (13%)
Current smokers	55 (8.8%)
Vaccination Status, *n* (%)	
Unvaccinated	234 (37%)
Vaccinated	393 (63%)
Number of Doses Received, *n* (%)	
0 Doses	234 (37%)
1 Dose	18 (2.9%)
2 Doses	82 (13%)
3 Doses	180 (29%)
4 Doses	113 (18%)
Vaccination Timing (among vaccinated), *n* (%) ^b^	
Vaccinated After Infection (Post-Acute) ^c^	226 (58%)
Vaccinated Before Infection (Prevention)	167 (42%)
Vaccine Regimen Strategy (among vaccinated), *n* (%) ^d^	
Homologous (Same vaccine)	246 (63%)
Heterologous (Mixed vaccine)	147 (37%)
long COVID Outcome, *n* (%)	
long COVID Symptoms Present	252 (40%)

*N*, total number of participants; *n*, number of participants in category; SD, Standard Deviation; BMI, Body Mass Index; ^a^ Percentages may not total 100% due to rounding or missing data. Specifically, smoking status data were unavailable for 102 participants (16%). ^b^ Percentages for Vaccination Timing and Vaccine Regimen Strategy were calculated based on the sub-cohort of vaccinated participants (*n* = 393), not the total study population. ^c^ Vaccinated Before Infection is defined as having received at least one vaccine dose ≥ 14 days prior to the first confirmed positive SARS-CoV-2 test. Vaccinated After Infection includes participants who received their first dose after the acute infection period. ^d^ Homologous refers to receiving the same vaccine type/brand for all doses; Heterologous refers to receiving a mix of different vaccine types/brands.

**Table 2:** Multivariable Bayesian Logistic Regression predicting long COVID (Prevention Cohort) ^b^.

Predictor ^c^	Adjusted OR ^a^	95% CI	*p*-Value
Vaccinated (vs. Unvaccinated)	0.81	0.45–1.42	0.453
Comorbidities (Any vs. None)	2.78	1.17–7.79	0.024 *
LoS (≥4 days)	1.82	1.03–3.27	0.038 *
Age (per year)	1.05	1.03–1.07	<0.001 *
Gender (Male vs. Female)	0.88	0.52–1.48	0.630
BMI (per unit)	1.02	0.99–1.06	0.236

OR, Odds Ratio; CI, Confidence Interval; BMI, Body Mass Index; LoS, Length of Hospital Stay. ^a^ Estimates were derived using a multivariable Bayesian logistic regression model (bayesglm) to account for potential data separation and small subgroup sizes. ^b^ Analysis is restricted to the “Prevention Cohort” (*n* = 389), comparing participants vaccinated ≥14 days prior to infection against those who remained unvaccinated. ^c^ Reference Groups: Unvaccinated (for Vaccination Status); No Comorbidities (for Comorbidities); Short LoS < 4 days (for Length of Stay); Female (for Gender). * Indicates statistical significance (*p* < 0.05).

**Table 3:** Subgroup Analysis: Association Between Vaccine Strategies and Long COVID (Prevention Cohort).

Predictor	Adjusted OR ^a^	95% CI	*p*-Value
Vaccination Strategy			
Unvaccinated (Reference)	1.00	—	—
Homologous	0.76	0.37–1.47	0.408
Heterologous	0.88	0.41–1.80	0.727
Clinical Covariates			
Comorbidities (Any vs. None)	2.78	1.17–7.79	0.024 *
Length of Stay (≥4 days)	1.82	1.03–3.27	0.038 *
Age (per year)	1.05	1.03–1.07	<0.001 *

OR, Odds Ratio; CI, Confidence Interval. ^a^ Estimates derived from a multivariable Bayesian logistic regression model adjusted for age, gender, BMI, comorbidities, and length of hospital stay. Homologous: Participants received the same vaccine brand/type for all doses. Heterologous: Participants received a “mix-and-match” combination of vaccine brands. * Indicates statistical significance (*p* < 0.05).

## Data Availability

Data will be made available on request.

## Abbreviations

aOR	Adjusted Odds Ratio
BMI	Body Mass Index
CI	Confidence Interval
COVID-19	Coronavirus Disease 2019
EDC	Electronic Data Capture
EHR	Electronic Health Records
LoS	Length of Hospital Stay
mRNA	Messenger Ribonucleic Acid
NHS	National Health Service
ONS	Office for National Statistics
OR	Odds Ratio
PASC	Post-Acute Sequelae of SARS-CoV-2 (clinical term for long COVID)
PCR	Polymerase Chain Reaction
SARS-CoV-2	Severe Acute Respiratory Syndrome Coronavirus 2
SD	Standard Deviation
STROBE	Strengthening the Reporting of Observational Studies in Epidemiology
WHO	World Health Organisation

## References

[1] Domènech-Montoliu S., Puig-Barberà J., Badenes-Marques G., Gil-Fortuño M., Orrico-Sánchez A., Pac-Sa M.R., Perez-Olaso O., Sala-Trull D., Sánchez-Urbano M., Arnedo-Pena A. (2023). Long COVID prevalence and the impact of the third SARS-CoV-2 vaccine dose: A cross-sectional analysis from the third follow-up of the Borriana cohort, Valencia, Spain (2020–2022). Vaccines.

[2] Chow K.N., Tsang Y.W., Chan Y.H., Telaga S.A., Ng L.Y.A., Chung C.M., Cheung P.P. (2024). The effect of pre-COVID and post-COVID vaccination on long COVID: A systematic review and meta-analysis. J. Infect..

[3] Bajema K.L., Dahl R.M., Prill M.M., Meites E., Rodriguez-Barradas M.C., Marconi V.C., Beenhouwer D.O., Brown S.T., Holodniy M., Lucero-Obusan C. (2021). Effectiveness of COVID-19 mRNA vaccines against COVID-19–associated hospitalisation—Five Veterans Affairs medical centres, United States, February 1–August 6, 2021. Morb. Mortal. Wkly. Rep..

[4] Al-Aly Z., Bowe B., Xie Y. (2022). Long COVID after breakthrough SARS-CoV-2 infection. Nat. Med..

[5] Ayoubkhani D., Bosworth M.L., King S., Pouwels K.B., Glickman M., Nafilyan V., Walker A.S. (2022). Risk of long COVID in people infected with severe acute respiratory syndrome coronavirus 2 after two doses of a coronavirus disease 2019 vaccine: A community-based, matched cohort study. Open Forum Infect. Dis..

[6] Byambasuren O., Stehlik P., Clark J., Alcorn K., Glasziou P. (2023). Effect of COVID-19 vaccination on long COVID: Systematic review. BMJ Med..

[7] Chiu N.C., Chi H., Tu Y.K., Huang Y.N., Tai Y.L., Weng S.L., Chang L., Huang D.T., Huang F.Y., Lin C.Y. (2021). To mix or not to mix? A rapid systematic review of heterologous prime-boost COVID-19 vaccination. Expert. Rev. Vaccines.

[8] Asante M.A., Michelsen M.E., Balakumar M.M., Kumburegama B., Sharifan A., Thomsen A.R., Korang S.K., Gluud C., Menon S. (2024). Heterologous versus homologous COVID-19 booster vaccinations for adults: Systematic review with meta-analysis and trial sequential analysis of randomised clinical trials. BMC Med..

[9] Trinh N.T., Jödicke A.M., Català M., Mercadé-Besora N., Hayati S., Lupattelli A., Prieto-Alhambra D., Nordeng H.M. (2024). Effectiveness of COVID-19 vaccines to prevent long COVID: Data from Norway. Lancet Respir. Med..

[10] Carazo S., Phimmasone J., Skowronski D.M., Giguère K., Ouakki M., Talbot D., Guay C.A., Sauvageau C., Brousseau N., De Serres G. (2025). Effectiveness of COVID-19 vaccination and prior infections to reduce long COVID risk during the pre-Omicron and Omicron periods. Clin. Infect. Dis..

[11] Green R., Marjenberg Z., Lip G.Y.H., Banerjee A., Wisnivesky J., Delaney B.C., Peluso M.J., Wynberg E., Abduljawad S. (2025). A systematic review and meta-analysis of the impact of vaccination on prevention of long COVID. Nat. Commun..

[12] Hernán M.A., Robins J.M. (2020). Causal Inference: What If.

[13] Patterson B.K., Francisco E.B., Yogendra R., Long E., Pise A., Rodrigues H., Hall E., Herrera M., Parikh P., Guevara-Coto J. (2022). Persistence of SARS-CoV-2 S1 protein in CD16+ monocytes in post-acute sequelae of COVID-19 (PASC) up to 15 months post-infection. Front. Immunol..

[14] Proal A.D., VanElzakker M.B. (2021). Long COVID or post-acute sequelae of COVID-19 (PASC): An overview of biological factors that may contribute to persistent symptoms. Front. Microbiol..

[15] Subramanian A., Nirantharakumar K., Hughes S., Myles P., Williams T., Gokhale K.M., Taverner T., Chandan J.S., Brown K., Simms-Williams N. (2022). Symptoms and risk factors for long COVID in non-hospitalised adults. Nat. Med..

